# Late-onset attention-deficit/hyperactivity disorder as a differential diagnosis of dementia: a case report

**DOI:** 10.1186/s12888-020-02949-7

**Published:** 2020-11-23

**Authors:** Hiroyuki Sasaki, Tadashi Jono, Ryuji Fukuhara, Seiji Yuki, Tomohisa Ishikawa, Shuken Boku, Minoru Takebayashi

**Affiliations:** 1 Department of Neuropsychiatry, Faculty of Life Science, Kumamoto University, 1-1-1 Honjo, Chuo-ku, Kumamoto-shi, Kumamoto 860-8556 Japan; 2 Institute for Clinical Research, National Hospital Organization Kure Medical Center Chugoku Cancer Center, Hiroshima, Japan

**Keywords:** Dementia, Early onset Alzheimer’s disease, Late-onset attention-deficit/hyperactivity disorder, Pre-elderly

## Abstract

**Background:**

Although adult attention-deficit/hyperactivity disorder has recently gained increased attention, few reports on attention-deficit/hyperactivity disorder in the pre-elderly or elderly have been published. Here, we present the case of a patient with attention-deficit/hyperactivity disorder who gradually developed dementia-like symptoms as she aged, which initially made her condition difficult to distinguish from early onset Alzheimer’s disease. This report illustrates that some types of attention-deficit/hyperactivity disorder may be misdiagnosed as dementia.

**Case presentation:**

The patient was a 58-year-old woman. Although she presented with a tendency for inattentiveness and forgetfulness since childhood, she did not have a history of psychiatric disorders prior to consultation. Around the age of 52 years, her inattentiveness and forgetfulness gradually progressed, and at 57 years of age, she became inattentive and forgetful that it interfered with her work and daily life. For example, she forgot meetings with important clients and transferred money to the wrong bank account; these failures resulted in poor management of her company. At home, she experienced increasing difficulties with remembering prior commitments with her family and misplacing items, which her family members noticed. With the encouragement of her family and employees, who worried that she was suffering from dementia, she visited our memory clinic, whereby she was suspected of having early onset Alzheimer’s disease. However, neuropsychological tests and brain imaging evaluations did not reveal any significant abnormalities. After dismissing various possible diagnoses, including dementia, other organic diseases, mood disorders, and delirium, we diagnosed her with attention-deficit/hyperactivity disorder. Treatment with 18 mg of methylphenidate was initiated, and significant improvements in her symptoms were observed within a few days; for example, she stopped losing her things, was able to concentrate for long durations, and could complete more tasks than she could before treatment. Since initiating treatment, she has returned to work and has been able to perform her daily activities without difficulty.

**Conclusions:**

This case supports that some patients with late-onset attention-deficit/hyperactivity disorder may gradually develop dementia-like symptoms during the pre-elderly and elderly stages of life. Therefore, clinicians should consider late-onset attention-deficit/hyperactivity disorder as a differential diagnosis of some types of dementias.

## Background

According to the Diagnostic and Statistical Manual of Mental Disorders, Fifth Edition (DSM-5), attention-deficit/hyperactivity disorder (ADHD) is a developmental disorder characterized by symptoms such as impulsivity, inattention, and hyperactivity [1]. Although the traditional clinical research focus is on ADHD in childhood, various researchers are now examining ADHD in adulthood [2–7]. It has been reported that the prevalence of ADHD in childhood and adulthood is approximately 5 and 2.5%, respectively. More than five out of nine diagnostic criteria must be fulfilled to diagnose ADHD in adulthood, in contrast to more than six items out of nine in childhood [1]. Similarly, although some differences in epidemiological characteristics or criteria exist, there is no clear evidence about the etiological differences in ADHD between children and adults. Despite these recent increased interest in adult ADHD, few reports on ADHD in the pre-elderly or elderly exist. Therefore, the aim of this report was to present the case of a patient who gradually developed dementia-like symptoms later in life (pre-elderly stages) but who was eventually diagnosed with late-onset ADHD.

## Case presentation

The patient was a 58-year-old woman with no history of psychiatric disorder before the age of 58. When she was younger (approximately 10 years old), despite tending to exhibit inattentiveness and forgetfulness, she obtained good grades in school, and these tendencies did not interfere with her daily life. Although her family noticed certain behaviors—such as inattentiveness and forgetfulness, which was manifested as hitting or misplacing things—they assumed that these tendencies were inherited, since other family members who exhibited similar issues were not diagnosed with psychiatric disease and did not require medical treatment. After graduating from high school, she worked at a bank. Although she was aware of a tendency to forget to take notes or misplace her documents at work, she never made any errors that caused serious repercussions and did not experience problems with her colleagues. After she retired from the bank at 52 years of age, she started her own business.

After starting her own business, she was much busier than she had been when she was simply an employee. Within a year, her attentional difficulties and memory issues gradually progressed, and at approximately 57 years of age, she became so inattentive and forgetful that it interfered with her work and family life at home. For example, she forgot meetings with important clients, transferred money to the wrong bank account, and these failures resulted in poor management of her company. At home, she experienced increasing difficulties with remembering prior commitments with her family and misplacing items, which her family members noticed. With the encouragement of her family and employees, who worried that she was suffering from dementia, she visited our memory clinic at 58 years of age.

**Fig. 1:**
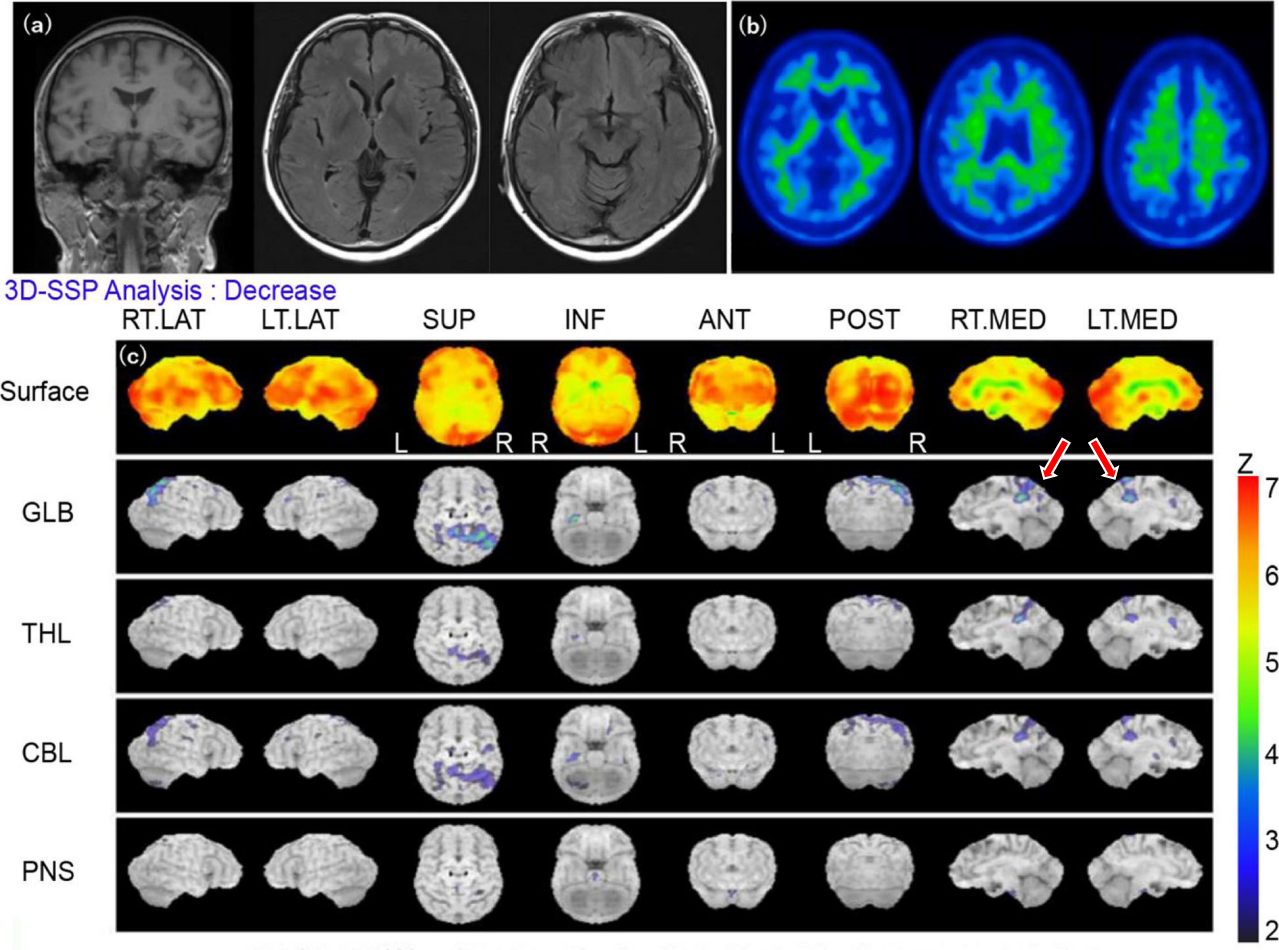
Brain imaging results of the patient with late-onset ADHD. **a** No particular change in brain MRI. **b** Negative finding of amyloid deposition in PiB-PET. **c** Slightly decreased blood flow in the posterior cingulate cortex on SPECT (contrast substance:^123^I-IMP). Abbreviations: ADHD, attention deficit hyperactivity disorder; ^123^I-IMP, N-isopropyl iodo-amphetamine; MRI, magnetic resonance imaging; PiB-PET, Pittsburgh compound B positron emission tomography; SPECT, single-photon emission computed tomography

Upon visiting the clinic, she reported that she perceived her increased forgetfulness was problematic since it was bothering the people around her; therefore, she feared having dementia and prepared herself to retire from her own company. She did not report experiencing reductions in her levels of desires or interests, and no depressive symptoms were noted during the medical interview. No neurological abnormalities, such as parkinsonism or pathological reflexes, were identified. Neuropsychological testing, such as Mini-Mental State Examination, Alzheimer’s Disease Assessment Scale-Cognitive subscale and Rivermead Behavioural Memory Test, indicated that her memory was intact. Laboratory tests, including blood counts (e.g., full blood count, electrolytes, urea and creatinine, vitamins B1 and B12, and folic acid), blood biochemistry, and electroencephalography, did not reveal any abnormalities (Table 1). Magnetic resonance imaging (MRI) of the brain did not reveal any clear cerebral parenchymal atrophy in the hippocampus or other regions, but single-photon emission computed tomography (SPECT) indicated mildly decreased blood flow in the posterior cingulate cortex. Pittsburgh compound B positron emission tomography (PiB-PET) did not show amyloid deposition in the cerebral cortex, and the phosphorylated tau/amyloid beta-42 ratio in the cerebrospinal fluid (CSF) was within the normal range (Fig. 1).

**Table 1:**
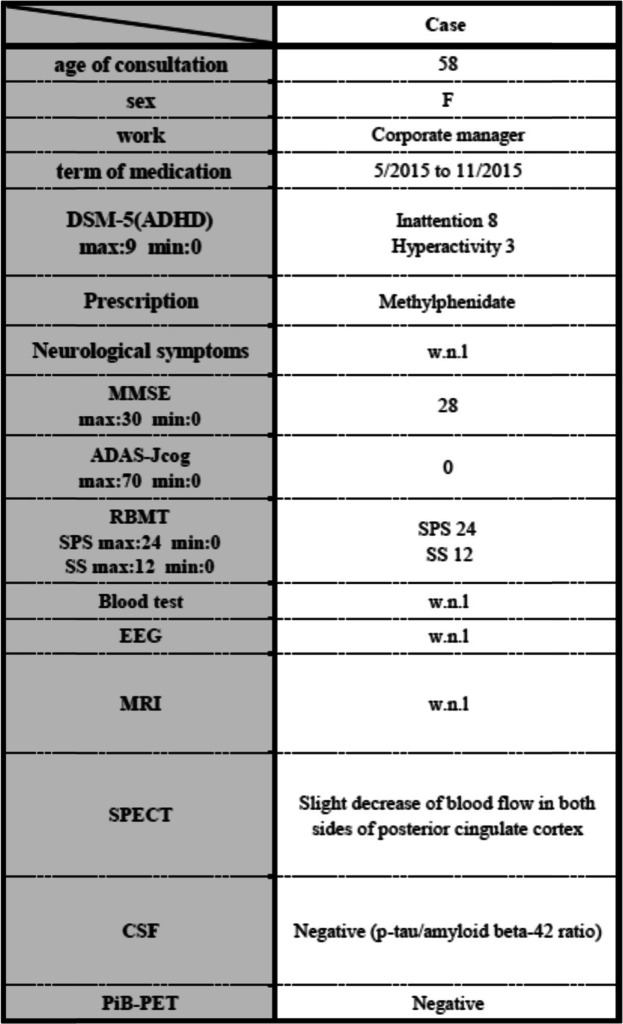
Summary of clinical information and examination results of the patient with late-onset ADHD

*Abbreviations*: *ADAS-Jcog* Alzheimer’s Disease Assessment Scale-cognitive subscale cognitive component-Japanese version, *CSF* Cerebrospinal fluid, *DSM-5* Diagnostic and Statistical Manual of Mental Disorders. 5th ed., *EEG* Electroencephalography, *F* Female, *MMSE* Mini-Mental State Examination, *MRI* Magnetic Resonance Imaging, *PiB-PET* Pittsburgh compound B positron emission tomography, *RBMT* Rivermead Behavioural Memory Test, *SPECT* Single-Photon Emission Computed Tomography, *w.n.l* within normal limits, *p-tau* phosphorylated tau

Given that her condition at work and at home fulfilled eight of the DSM-5 diagnostic criteria for inattentiveness and three for hyperactivity/impulsivity, we diagnosed her with ADHD, and treatment with 18 mg of methylphenidate was initiated and continued this dose. Within a few days after starting the methylphenidate administration, pronounced improvements in her symptoms were observed; for example, she stopped losing her things, was able to concentrate for long durations, and could complete more tasks than she could prior to treatment, which surprised her family and subordinates. Thus, she no longer met the DSM-5 diagnostic criteria for ADHD (Table 2). Although she thought she would have to retire from her business, she instead was able to return to work without issue. We conducted a follow up for 6 months with administration of 18 mg methylphenidate; during this period, her initial symptoms did not reappear.

**Table 2:**
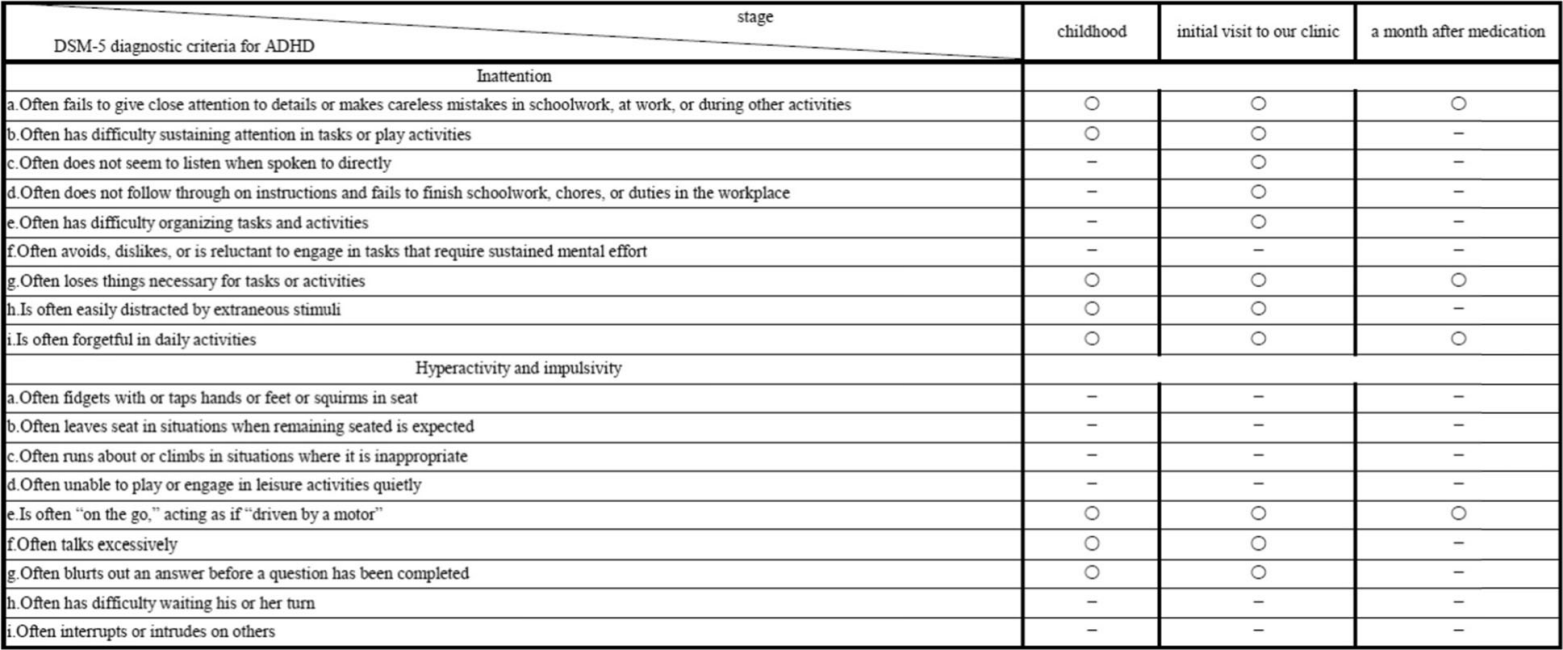
Time course of the clinical characteristics based on the DSM-5 criteria for ADHD of the patient with late-onset ADHD

Circles and lines indicate fulfillment and non-fulfillment of the criterion, respectively

## Discussion and conclusions

The present report described the case of a patient whose memory impairment and attentional difficulty became more pronounced after 52 years of age than before and gradually progressed. She was initially suspected to have early onset Alzheimer’s disease (EOAD) in our clinic because she was 58 years old, which is the onset age of this disease. Furthermore, her clinical course resembled that of EOAD as patients often present with symptoms of forgetfulness and inattention rather than behavioral and psychological symptoms of dementia, such as visual hallucinations and personality changes. Additionally, EOAD onset is often noticed by surrounding people because of the disturbed social activity caused by these symptoms [8]. Although neuropsychological tests and brain MRI revealed no abnormalities, SPECT indicated mildly decreased blood flow in the posterior cingulate cortex. Because the posterior cingulate cortex is the first region to exhibit blood flow decreases in AD [9, 10], we also performed PiB-PET and measured the total tau/Aβ42 ratio in the CSF, which are believed to be sensitive tests for AD pathology [11]. According to these results, we finally ruled out the possibility of EOAD.

Although she had exhibited the tendency of inattentiveness and forgetfulness since childhood, a retrospective review of her medical records revealed that these symptoms never met the diagnostic criteria for ADHD. But interestingly, the symptoms that were present upon visiting our clinic fulfilled the DSM-5 diagnostic criteria for ADHD. Briefly, she met the diagnostic criteria for ADHD for the first time at 58 years of age (Table 2). As in this case, patients who meet the diagnostic criteria for ADHD for the first time in adulthood have recently been considered to have late-onset ADHD. There have been several reports on late-onset ADHD [12–15]. However, these reports have been limited to young adults, and some details about late-onset ADHD in older adults are considered unknown tasks [12], which our report details as “very” late-onset ADHD in older adults. Existing reports in the literature of cases of late-onset ADHD can be divided into those involving patients showing the “new onset” of ADHD [13, 14] and those involving patients showing a “manifestation” of the disease [12, 15]. Although our case corresponds to the latter, further study is needed to clarify whether there are indeed two types of late-onset ADHD (“new onset” and “manifestation”).

Our patient could return to work owing to the therapeutic effect of methylphenidate. However, methylphenidate may exert a clinical effect on diseases other than ADHD. For example, a study indicated that methylphenidate improved some symptoms of frontotemporal degeneration (FTD) [16]. Therefore, our patient’s therapeutic effect is not directly connected with ADHD. Although we followed this patient for 6 months and observed no relapse of symptoms, it may be ideal to follow the patient for a few more years to rule out FTD and other dementias (including mild cognitive impairment). On the other hand, as mentioned above, it was true that her forgetfulness and carelessness became obvious in recent years, causing severe damage to the company she established, and she satisfied the diagnostic criteria for ADHD. Her symptoms were accounted for by ADHD but not by other diseases, such as dementia.

Collectively, the details from our case suggests the existence of very late-onset ADHD. Previous studies about late-onset ADHD have been limited to young adults, and this is the first report on older adults. Some patients with very late-onset ADHD may be misdiagnosed with dementia because their symptoms arise in the pre-elderly or elderly stages of life. Distinguishing between very late-onset ADHD and dementia may be useful in preventing misdiagnosis. However, this is difficult to clarify solely from this case report; thus, further studies are needed to examine very late-onset ADHD.

## Data Availability

Not applicable.

## Abbreviations

ADHD: Attention-deficit/hyperactivity disorder
DSM-5: Diagnostic and Statistical Manual of Mental Disorders, Fifth Edition
MRI: Magnetic resonance imaging
SPECT: Single-photon emission computed tomography
PiB-PET: Pittsburgh compound B positron emission tomography
CSF: Cerebrospinal fluid
FTD: Frontotemporal dementia
